# Modelling the ecological dynamics of mosquito populations with multiple co-circulating *Wolbachia* strains

**DOI:** 10.1038/s41598-022-25242-x

**Published:** 2022-12-02

**Authors:** Samson T. Ogunlade, Adeshina I. Adekunle, Emma S. McBryde, Michael T. Meehan

**Affiliations:** 1 Australian Institute of Tropical Health and Medicine, James Cook University, Townsville, QLD Australia; 2 College of Medicine and Dentistry, James Cook University, Townsville, QLD Australia; 3 Department of Defence, Defence Science and Technology Group, Melbourne, VIC Australia

**Keywords:** Applied mathematics, Viral infection, Disease model

## Abstract

*Wolbachia* intracellular bacteria successfully reduce the transmissibility of arthropod-borne viruses (arboviruses) when introduced into virus-carrying vectors such as mosquitoes. Despite the progress made by introducing *Wolbachia* bacteria into the *Aedes aegypti* wild-type population to control arboviral infections, reports suggest that heat-induced loss-of-*Wolbachia*-infection as a result of climate change may reverse these gains. Novel, supplemental *Wolbachia* strains that are more resilient to increased temperatures may circumvent these concerns, and could potentially act synergistically with existing variants. In this article, we model the ecological dynamics among three distinct mosquito (sub)populations: a wild-type population free of any *Wolbachia* infection; an invading population infected with a particular *Wolbachia* strain; and a second invading population infected with a distinct *Wolbachia* strain from that of the first invader. We explore how the range of possible characteristics of each *Wolbachia* strain impacts mosquito prevalence. Further, we analyse the differential system governing the mosquito populations and the *Wolbachia* infection dynamics by computing the full set of basic and invasive reproduction numbers and use these to establish stability of identified equilibria. Our results show that releasing mosquitoes with two different strains of *Wolbachia* did not increase their prevalence, compared with a single-strain *Wolbachia*-infected mosquito introduction and only delayed *Wolbachia* dominance.

## Introduction

*Wolbachia* infection in arthropods, in particular, *Aedes aeqypti* mosquitoes is capable of inhibiting the transmission of arboviruses such as Zika (ZIKV), Chikungunya (CHIKV) and dengue viruses (DENV)^1–4^. These arboviruses have been estimated to infect over 390 million people annually causing significant global health problems^1,5–7^.

*Aedes aegypti* mosquitoes do not naturally host the intracellular biosymbiotic *Wolbachia* bacteria, but can be infected through microinfection^3^. The *Wolbachia*-based technique of arboviral vector control is predominantly aimed at two mechanisms: distrupting arboviral transmission between vectors and hosts; and suppressing the vector population^8^. Some *Wolbachia* features regulating the success of these mechanisms include immune system preactivation in the vectors, induction of cytoplasmic incompatibility (CI) rendering offspring unviable, imperfect maternal transmission of *Wolbachia*, loss of *Wolbachia* infection (LWI) due to high temperature, and superinfection by a second *Wolbachia* strain^9–12^. Based on these features, there are some tradeoffs exhibited by different *Wolbachia* strains, i.e., some strains induce CI (which is good) but also have LWI due to high temperature (which is bad) and vice versa^13,14^.

**Figure 1:**
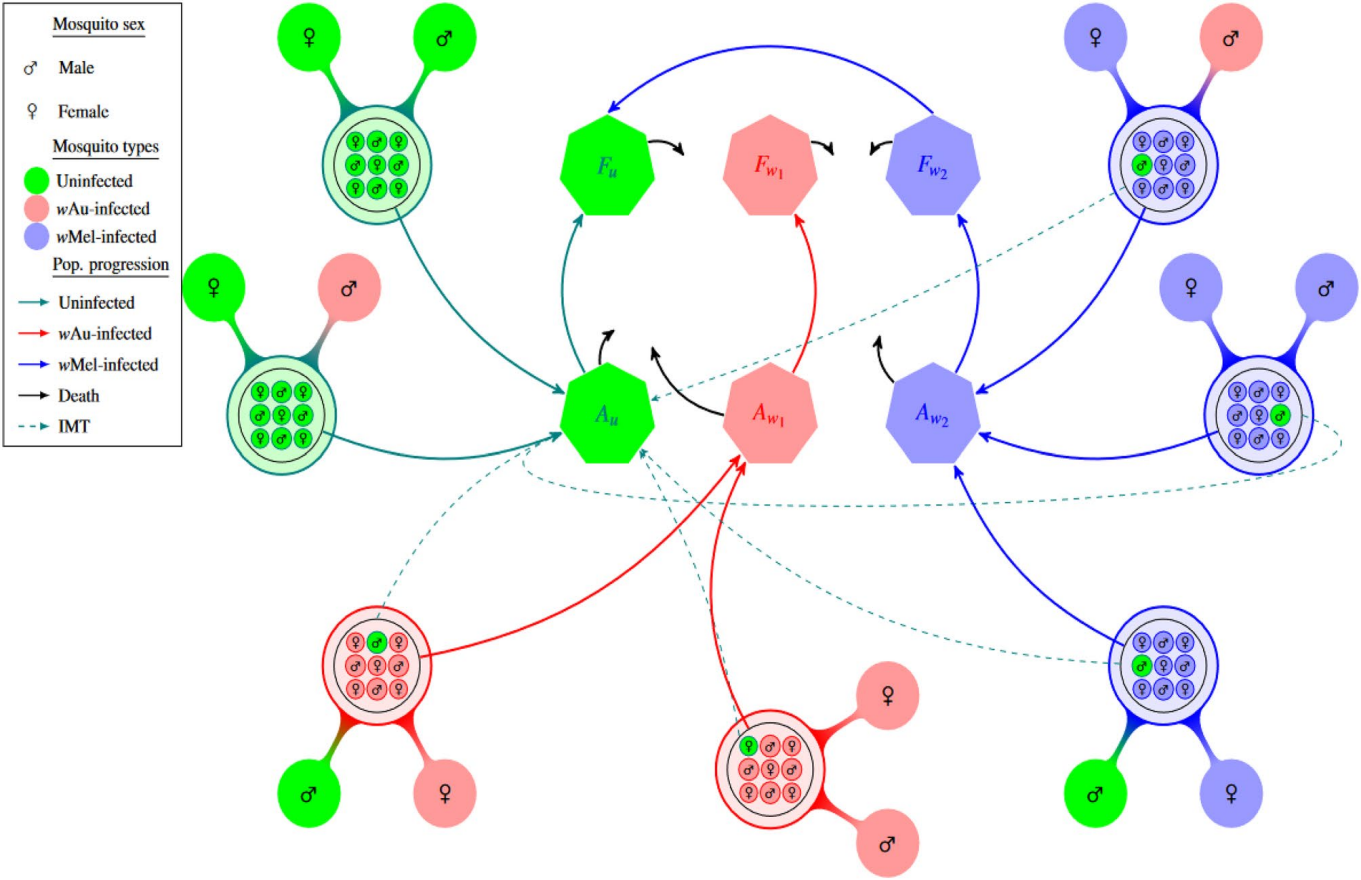
Model schematic of Mosquito-*Wolbachia* dynamics between uninfected mosquitoes *u* and ***Wolbachia***-infected mosquitoes with strains $\mathbf {w_1}$ (***w***Au-like) and $\mathbf {w_2}$ (***w***Mel-like). The green, red, and blue represent the uninfected, *w*Au-*Wolbachia*-infected and *w*Mel-*Wolbachia* infected mosquito populations respectively. The lines (solid and dashed) represent the population progression where the dashed lines indicate the imperfect maternal transmission (IMT). The black arrows represent deaths. The cytoplasmic incompatibility (CI) induction which inhibits the production of offspring has been adjusted where required. $A\longrightarrow$ Aquatic (eggs, larvae and pupae) mosquitoes and $F\longrightarrow$ Adult mosquitoes.

Presently, the *w*Mel-*Wolbachia* strain is commonly used in the field, with releases in Australia^15^, Indonesia^16^, Brazil^17^, Colombia^18^, the United States of America and China^19^. The *w*AlbB *Wolbachia* strain was later introduced in Malaysia^20^, Thailand^21^, Taiwan^22^, India^14^ and *w*MelPop in Vietnam^23^, while other strains are yet to be field-tested. Single-strain *Wolbachia* experimental studies have shown that most crosses between *Wolbachia*-infected arthropods and wild-type mosquitoes induce unidirectional CI, that is, loss of fertility of a wild-type female mating with a *Wolbachia*-infected male mosquito, but not the reverse^2,24–26^. In addition, most *Wolbachia*-infected mosquitoes greatly lose their infection under high temperatures^12,27^ except those infected with the CI-inducing *w*AlbB and *w*Au-*Wolbachia* strains, which does not induce CI^3,10,14^. For double-strain *Wolbachia* experimental studies, CI is typically bidirectional, that is, any mismatch in *Wolbachia* strain among mating vectors results in infertility; however, CI does not affect crosses involving *w*Au-*Wolbachia*-infected males with other *Wolbachia*-infected females^3,6,28,29^, opening up a tantalising possibility of two different strains of *Wolbachia*-infected mosquitoes co-existing (Fig. 1).

Most existing *Wolbachia* modelling studies have only analysed single-strain *Wolbachia* dynamics in arthropod vectors^11,30–38^. Meanwhile, those studies that have modelled, discussed or compared the existence of multiple *Wolbachia* subpopulations^1,4,6,28,39^, only consider *Wolbachia* strains with the same CI induction and heat-susceptibility characteristics (e.g. *w*Mel and *w*MelPop strains). Some recent studies compared two different and separate *Wolbachia* strains: *w*Au and *w*Mel^40^, *w*AlbB and *w*Mel^1^, and *w*AlbB/*w*MelCS and *w*Mel^6^. The authors in^40^ investigated the use of vaccination and two *Wolbachia* strains (*w*Au and *w*Mel) to reduce dengue incidence and showed that although both strains can be used to mitigate dengue, *w*Au performed better than *w*Mel. Flores *et al*.^6^, showed that the transmission potential of *Wolbachia*-infected mosquitoes was greatly reduced for *w*MelCS and *w*AlbB compared to *w*Mel. In addition, Xue *et al*.^1^, showed that *w*Mel, *w*AlbB and *w*MelPop *Wolbachia* strains can effectively reduce arboviral transmission. However, of the three, *w*MelPop has the highest fitness cost to the mosquito and would require a sufficiently large number of *w*MelPop-infecfed mosquitoes to be introduced in order to establish themselves in the *Wolbachia*-free mosquito population^1^.

Keeling *et al* developed continuous-time models that captured the dynamics of mosquitoes with both one and two co-circulating *Wolbachia* strains^28^. They showed that in a single-strain model, a *Wolbachia*-infected population cannot invade a wild-type mosquito population unless the proportion of infected mosquitoes is high enough to break through the critical infection threshold—an example of the Allee effect^41^. For two strains, they showed that the models exhibit the founder control effect^28^: either of the strains could invade from low density levels if the other strain is present. Further, in a mixed mosquito population with two *Wolbachia* strains, the authors showed the coexistent equilibrium is unstable as one strain will knock out the other depending on the parameters and densities defining the strains. That is, a *Wolbachia* dominant strain defined by *Wolbachia*-favourable parameters will outperform the other^28^. However, moving from a homogeneous to a spatially heterogeneous system, the two *Wolbachia* strains may coexist locally. This could be established only by the inflow of two different *Wolbachia* strains in the areas defined between bounded regions of different patches of *Wolbachia*-infected mosquito habitats^28^. Similar studies investigated the introduction of *Wolbachia*-infected mosquitoes with different mortality and fertility rates and showed that *Wolbachia*-infected mosquitoes will not dominate the wild-mosquito population if the efficacy of the vertical (maternal) transmission is less than 75%^42^. In addition, *Wolbachia* infection was predicted to easily spread among the wild-type population for higher transmission rates^43^. Two recent modelling studies^44,45^ considered the spread of *Wolbachia* infection in mosquitoes via delay differential equations. They showed that *Wolbachia* infection will established itself and dominate the wild-type mosquito population if the *Wolbachia* release level surpasses the basic reproductive number of the *Wolbachia*-infected mosquitoes^44,45^. Another recent modelling study^39^ showed that the introduction of multiple *Wolbachia* strains could be more efficient than a single-strain introduction depending on the number, frequency and fitness cost of *Wolbachia* introductions. For low fitness cost imposed by *Wolbachia*, the single-strain introduction is efficient in achieving *Wolbachia* dominance with more frequent introductions of the same strain. In this work, we want to assess whether two-*Wolbachia*-strain introduction is better than one with respect to the *Wolbachia* loss and CI attributes of each strain.

As mentioned above, the *w*Au and *w*AlbB strains are heat-resistant however, *w*Au does not induce CI. On the other hand, the *w*Mel strain does induce CI but is more heat sensitive. The *w*Mel strain is effective at reducing transmission potential (quantified by the presence or absence of dengue virus in saliva-inoculated mosquitoes) but not as effective as *w*AlbB and *w*MelCS^3,6^. Therefore, the two-strain model involving different CI and heat loss features of *Wolbachia* strains such as *w*Au and *w*Mel, *w*Au and *w*AlbB or *w*AlbB and *w*Mel has the potential to demonstrate synergies of these strains. Such two-strain models have to the authors knowledge not previously been developed.

In this study, we develop a general two-strain *Wolbachia* model that could account for any two particular *Wolbachia* strains. We then adjust the model to capture two particular *Wolbachia* strains with contrasting high temperature and CI induction behaviours (Fig. 1). The general *Wolbachia* model is an extension of the single-strain *Wolbachia* transmission model considered in^13^, which explored the dynamics between crosses of *w*Au and wild-type, and *w*Mel and wild-type mosquitoes. The results in^13^ showed that despite a lack of CI-induction, the single *w*Au strain could be more effective than *w*Mel in sustaining *Wolbachia* infection as its *Wolbachia* infection retention feature could outweigh that of CI-inducing strains such as *w*Mel, which is susceptible to high temperature. In our adjusted two-strain *Wolbachia* model, we consider both uni-and bi-directional CI together with temperature-induced *Wolbachia* loss where necessary. We also consider the effect of imperfect maternal transmission in the model. We analyse the resulting differential system by computing the basic and invasive reproductive numbers and explore the two-strain *Wolbachia* model’s practicality for *Wolbachia* dominance.

## Methods

### Model formation

In this study, we formulate a general two-strain *Wolbachia* model which accommodates the combined interaction of two *Wolbachia* strains with arbitrary characteristics. The total mosquito population is categorised into three subpopulations namely the wild-type, uninfected mosquitoes (*u*), mosquitoes infected with the first *Wolbachia* strain ($w_1$) (e.g., *w*Au), and mosquitoes infected with the second *Wolbachia* strain ($w_2$) (e.g., *w*Mel/*w*AlbB) (see Supplementary figure S2). The Supplementary figure S2 shows the population progression from matings of male and female adult mosquitoes (from nine possible mating pairs) to offspring, regulated by CI effects, imperfect maternal transmission (IMT) and *Wolbachia* infection loss for a general two-strain *Wolbachia* model. As a particular example, that includes the effects of both uni and bidirectional CI and IMT, Fig. 1 depicts the population progression following the feasible matings between *w*Au-like and *w*Mel-like adult mosquitoes. Other schematics showing the two-strain *Wolbachia* combinations of *w*Au and *w*AlbB, and *w*Mel and *w*AlbB are shown in the Appendices section (Supplementary Figures S3 and S4).

**Table 1:** Mosquito-*Wolbachia* Model Parameters.

Parameters	Description	Values	Dimension
**Population size**			
$A_{i}$	Number of aquatic stage (egg, larvae, pupae) mosquitoes with infection status *i*		–
$F_{i}$	Number of adult female mosquitoes with infection status *i*		–
$M_{i}$	Number of adult male mosquitoes with infection status *i*		–
*K*	Carrying capacity of the aquatic stage mosquitoes		Aquatic mosquitoes
**Proportions**			
$\eta _{ij}$	Proportion of eggs (offspring) with infection *i* produced from female parent with infection *i* mating with male parent with infection *j*	0-1^2,30^	–
$1-\eta _{ij}$	Proportion of uninfected eggs (offspring) produced from female parent with infection *i* mating with male parent with infection *j*	0-1^2,13^	–
$\phi _{ij}$	Uni- or bidirectional CI effectiveness for adult female mosquito with infection *i* mating with adult male mosquito with infection *j*	0 or 1^3,10^	–
**Per-capita rates**			
$\rho _u$	Egg-laying rate of uninfected female mosquitoes	13^30,46^	Eggs/day
$\rho _i$	Egg-laying rate of female mosquitoes with infection status *i*	10-11^30,46^	Eggs/day
$\sigma _i$	Rate of *Wolbachia* infection loss for mosquitoes with infection status *i*	0-0.02^13^	Day$^{-1}$
$\tau _{i}$	Maturation rate for aquatic stage mosquitoes of *i* into adulthood	0.11^2,46^	day$^{-1}$
$\mu _{A_i}$	Mortality rate for aquatic stage mosquitoes of infection type i	0.02^31^	day$^{-1}$
$\mu _{i}$	Mortality rate for reproductively mature (adult) mosquitoes with infection status *i*	0.043-0.082^3,10,47^	day$^{-1}$

Let *F*, *M* and *A* be the total number of female, male and aquatic mosquitoes respectively:

1 $$\begin{aligned} F=\sum _{k\epsilon \{u,w_1,w_2\}} F_k, M= \sum _{k\epsilon \{u,w_1,w_2\}} M_k, A= \sum _{k\epsilon \{u,w_1,w_2\}} A_k \end{aligned}$$

where subscripts denote the infection status of each subpopulation. Equation (1) describes the total sum of uninfected, $w_1$-*Wolbachia*-infected and $w_2$-*Wolbachia*-infected mosquitoes for adult female, male and aquatic individuals. In what follows, we assume $M=F$ so as to simplify the system following observational studies that recorded no significant difference in male to female (*Aedes aegypti* and *Aedes albopictus*) mosquito ratio^48,49^. The mathematical equations describing the two-strain *Wolbachia* transmission dynamics together with the mosquitoes’ reproductive rates for the general case are written as:

2 $$\begin{aligned} \frac{dA_{u}}{dt}= & \xi _u\left( 1-\frac{A}{K} \right) -(\tau _{u} +\mu _{A_u}) A_u,\nonumber \\ \frac{dF_{u}}{dt}= & \frac{\tau _{u}}{2}A_u +\sum _{j\epsilon \{w_1,w_2\}} \sigma _j F_{j} - \mu _u F_u,\nonumber \\ \frac{dA_{i}}{dt}= & \xi _i\left( 1-\frac{A}{K} \right) - (\tau _{i} +\mu _{A_{i}}) A_{i},\nonumber \\ \frac{dF_{i}}{dt}= & \frac{\tau _{i}}{2}A_{i} - (\mu _{i} + \sigma _i)F_{i}, \end{aligned}$$

where, $i\epsilon \{w_1, w_2\}$ represents the infection status/type, the carrying capacity (*K*) is a derived parameter quantifying the availability of the mosquito ovipositional breeding sites in a given location where aquatic stage mosquitoes would mature into adulthood. For our purposes, *K* provides an upper bound on the size of the aquatic stage mosquito population in a particular location. The differential equations in (2) represent the dynamics of the compartments for the $A_u, F_u, A_i, F_i$ which yield the number of uninfected aquatic stage, uninfected adult, *i*-infected aquatic stage and *i*-infected adult mosquitoes respectively. Therefore,

3 $$\begin{aligned} \xi _u= & \frac{\rho _{u} F_u\sum _{k\epsilon \{u,w_1,w_2\}}\left( [1-\phi _{uk}] F_{k}\right) + \sum _{j\epsilon \{w_1,w_2\}}\left( \rho _{j}F_j \sum _{k\epsilon \{u,w_1,w_2\}}\left( [1-\eta _{jk}] [1-\phi _{jk}]F_k\right) \right) }{F},\nonumber \\= & \frac{\rho _{u}F_u (F_u + F_{w_1}) + \rho _{w_1}F_{w_{1}}\left( [1-\eta _{w_1u}]F_u + [1-\eta _{w_1w_1}] F_{w_{1}}\right) + \rho _{w_2}F_{w_{2}}\left( [1-\eta _{w_2u}] F_{u}+ [1-\eta _{w_2w_2}] F_{w_{2}} + [1-\eta _{w_2w_1}] F_{w_1}\right) }{F},\nonumber \\ \xi _i= & \frac{ \rho _{i}F_{i}\sum _{k\epsilon \{u,w_1,w_2\}}\left( \eta _{ik}[1-\phi _{ik}] F_{k}\right) }{F},\nonumber \\= & {\left\{ \begin{array}{ll} \xi _{w_1} = \frac{ \rho _{w_1} F_{w_1} (\eta _{w_1w_1} F_{w_1} + \eta _{w_1u} F_u)}{F}\\ \xi _{w_2} = \frac{\rho _{w_2} F_{w_{2}} (\eta _{w_2w_2} F_{w_{2}} + \eta _{w_2u} F_u + \eta _{w_2w_1} F_{w_1})}{F}, \end{array}\right. } \end{aligned}$$

where $\rho _{j}F_j \sum _{k\epsilon \{u,w_1,w_2\}}\left( [1-\eta _{jk}] [1-\phi _{jk}]F_k\right)$ is the proportion of mosquito offspring that are generated from the mating combination of a female mosquito with infection status *i* and any other (infected or uninfected) male mosquito and accounting for CI as necessary. The $\xi _u$ and $\xi _i$ in equation (3) represent the total reproductive rates (measured as eggs per day) across all breeding combinations for uninfected and *i*-infected aquatic mosquitoes respectively.

Each of the model parameters appearing in equations (2) and (3) are described in Table 1. To rescale the above differential system with respect to the total population size using *K*, we have that $\sum _{k}A_k$, the sum of the aquatic stage mosquitoes with infection $k\epsilon \{u, w_1, w_2\}$ is less than or equal to the carrying capacity, which yields

$$\begin{aligned} \sum _{k}A_k\le K. \end{aligned}$$

This implies that

$$\begin{aligned} A_i\le K. \end{aligned}$$

From system (2), we also have the constraints $F_i \le \dfrac{\tau _i K}{2(\sigma _i + \mu _i)}$, and $F_u \le \dfrac{K}{2\mu _u}\left( \tau _u + \sum _{j}\dfrac{\sigma _j \tau _j}{\sigma _j + \mu _j}\right) .$ Combining the above results yields

$$\begin{aligned} \sum _{k \in \{u, w_1, w_2\} }\left( A_k(t) +F_k(t)\right)\le & K\left( 1+\frac{1}{2}\left( \dfrac{\tau _u}{\mu _u} + \sum _{ j \in \{w_1, w_2\}} \dfrac{\tau _{j}}{(\mu _{j} +\sigma _j)}\left( 1+\dfrac{\sigma _j}{\mu _u}\right) \right) \right) = \alpha K \end{aligned}$$

where $$\alpha = 1+\dfrac{1}{2}\left( \dfrac{\tau _u}{\mu _u} + \sum _{j} \dfrac{\tau _{j}}{(\mu _{j} +\sigma _j)}\left( 1+\dfrac{\sigma _j}{\mu _u}\right) \right)$$.

Given the above, it is straightforward to show that the closed set

$$\begin{aligned} \Omega = \left\{ (A_u, F_u, A_{w_1}, F_{w_1}, A_{w_2}, F_{w_2}) \in {\mathbb {R}}_+^6 \, | \, \sum _{k}\left( A_k(t) +F_k(t)\right) \le \alpha K \right\} \end{aligned}$$

is the feasible region for the system dynamics and is positively invariant^30^.

Rescaling each of the state variables in terms of the quantity $\alpha K$ gives

4 $$\begin{aligned} \frac{dA_{u}}{dt}= & \xi _u\left( 1-\alpha A \right) -(\tau _{u} +\mu _{A_u}) A_u,\nonumber \\ \frac{dF_{u}}{dt}= & \frac{\tau _{u}}{2}A_u +\sum _{j\epsilon \{w_1,w_2\}} \sigma _j F_{j} - \mu _u F_u,\nonumber \\ \frac{dA_{i}}{dt}= & \xi _i\left( 1-\alpha A \right) - (\tau _{i} +\mu _{A_{i}}) A_{i},\nonumber \\ \frac{dF_{i}}{dt}= & \frac{\tau _{i}}{2}A_{i} - (\mu _{i} + \sigma _i)F_{i}. \end{aligned}$$

Therefore, the general *Wolbachia* model in equation (2) in terms of population proportion becomes equation (4). Hence, in the scaled system (4), the sum of the state variables has an upper bound of 1. That is,

$$\begin{aligned} \sum _{k\epsilon \{u,w_1,w_2\}}(A_k + F_k) \le 1. \end{aligned}$$

## Results

### Model equilibria

The main three features of our general, two-strain *Wolbachia* model (4) are: (i) loss of infection at high temperatures; (ii) cytoplasmic incompatability; and (iii) imperfect maternal transmission. With these *Wolbachia* characteristics, we want to calculate the system equilibria and determine the conditions for their stability. Theoretically, we investigate six possible equilibrium points: a mosquito-free equilibrium; a wild-type (infection-free) mosquito-only equilibrium; a single-strain *Wolbachia*-only equilibrium; a coexistent wild-type and single-strain *Wolbachia*-infected equilibrium; a coexistent two different *Wolbachia* strains equilibrium; and finally, a multi-strain equilibrium where all three mosquito subpopulations coexist. We find that the first four of these are possible, but the last two are not.

To facilitate our equilibrium analysis, we first calculate a set of basic and invasive reproductive numbers for each mosquito subpopulation, both in the presence and absence of other mosquitoes. The set of invasive reproductive numbers represent the number of new mosquitoes of a particular type (specified by the first index, prior to the | separator) that would be generated by a single mosquito of that type when introduced into various mosquito population backgrounds (specified by the second index, following the | separator). For example, the quantity $R_{0i|u}$ is the average number of new mosquitoes with infection *i* that would be produced by a single *i*-infected mosquito throughout its lifespan, when it is introduced into a background of uninfected mosquitoes. Whereas, $R_{0u|i}$ is the average number of new uninfected mosquitoes generated by the introduction of an uninfected mosquito into an endemic mosquito population with infection status *i*, throughout its lifetime. An exception to this convention are the quantities $R_{0u}$ and $R_{0i}$ which respectively give the number of the new uninfected and infected mosquitoes generated (per index) when no (or few) background mosquitoes are present. Following this definition we see that each of the $R_0$ terms represent ratios and are therefore dimensionless. Hence, $R_{0u}$ and $R_{0i}$ are derived as:

$$\begin{aligned} R_{0u}= & \frac{\rho _u (1-\phi _{uu}) \tau _u}{2\mu _u (\mu _{A_u}+\tau _u)}=\frac{\rho _u \tau _u}{2\mu _u (\mu _{A_u}+\tau _u)},\\ R_{0i}= & \frac{\rho _{i}\eta _{ii} (1-\phi _{ii}) \tau _{i}}{2(\mu _{i}+\sigma _{i})(\mu _{A_i}+\tau _{i})}= \frac{\rho _{i}\eta _{ii} \tau _{i}}{2(\mu _{i}+\sigma _{i})(\mu _{A_i}+\tau _{i})}, \end{aligned}$$

where we have substituted in the values $\phi _{uu} = \phi _{ii} = 0$. This is because CI does not affect the matings between mosquitoes with the same infection status. In the event of perfect maternal transmission (i.e., $\eta _{ii} = 1$) and infection retention ($\sigma _i = 0$), the basic reproductive numbers of the *Wolbachia* strains ($R_{0i}$) become analogous to the simpler expression given for the wild-type subpopulation ($R_{0u}$).

For the mosquito-free equilibrium, we find that it is ecologically unrealistic, however, we numerically showed that if $\max {[R_{0u},R_{0i}]}<1$ the mosquito populations will go extinct, otherwise, they will persist (see Supplementary file and Figure S1).

Next, we will establish the single (*Wolbachia*-free and *Wolbachia*-infected) mosquito population equilibrium points and determine the conditions under which they are stable.

#### *Wolbachia*-free mosquitoes only

For the two-strain model (4), we first consider the existence and stability conditions for the persistence of *Wolbachia*-free mosquitoes only. We find that the infection-free equilibrium point is

$$\begin{aligned} e_u = ({\bar{A}}_u^u, {\bar{F}}_u^u, {\bar{A}}_{w_1}^u, {\bar{F}}_{w_1}^u, {\bar{A}}_{w_2}^u, {\bar{F}}_{w_2}^u) = \left( \dfrac{1}{\alpha }\left[ 1-\dfrac{1}{R_{0u}}\right] ,\frac{\tau _{u}}{2\alpha \mu _{u}}\left[ 1-\dfrac{1}{R_{0u}}\right] ,0,0,0,0\right) \end{aligned}$$

where the overbar and superscript denote that these state variables are equilibrium values. The equilibrium point $e_u$ exists if and only if $R_{0u}>1$.

Using the next generation matrix method, we obtain the invasive reproductive numbers $R_{0i|u}$ which are the average number of offspring that will be $i\epsilon \{w_1, w_2\}$ *Wolbachia*-infected after introducing a single infected adult into a completely susceptible (wild-type) mosquito population. We find that for any two competing strains $\phi _{iu}=\phi _{ii}=0$ (no CI induction between $F_iM_u$ and $F_iM_i$), such that $\xi _{u}\rightarrow \rho _u(1-\phi _{uu})F_u = \rho _u F_u$ and $\xi _{i}\rightarrow 0$. In this case, we find

5 $$\begin{aligned} R_{0i|u}= & \frac{R_{0i}\eta _{iu}}{R_{0u}\eta _{ii}}, \end{aligned}$$

where $R_{0i|u}$ in equation (5) is the invasive reproductive number with respect to infected mosquitoes with infection *i*. To establish the stability of $e_u$, we evaluate the Jacobian at this equilibrium point, $(J^{e_u})$, and then calculate the characteristic equation $|J^{e_u}-\lambda I|=0$, which gives:

$$\begin{aligned} (\lambda ^2 + a_1\lambda +a_2) (\lambda ^2 + a_3\lambda + a_4) (\lambda ^2 + a_5\lambda + a_6)=0 \end{aligned}$$

where

$$\begin{aligned} a_1= & \mu _u + (\mu _{Au}+\tau _u) R_{0u}\\ a_2= & \mu _u (\mu _{Au}+\tau _u) (R_{0u}-1)\\ a_3= & \mu _{w_1} + \sigma _1 + \mu _{Aw_1}+\tau _{w_1}\\ a_4= & (\mu _{w_1} + \sigma _1) (\mu _{Aw_1}+\tau _{w_1}) (1-R_{0w_1|u})\\ a_5= & \mu _{w_2} + \sigma _2 + \mu _{Aw_2}+\tau _{w_2}\\ a_6= & (\mu _{w_2} + \sigma _2) (\mu _{Aw_2}+\tau _{w_2}) (1-R_{0w_2|u}). \end{aligned}$$

**Figure 2:**
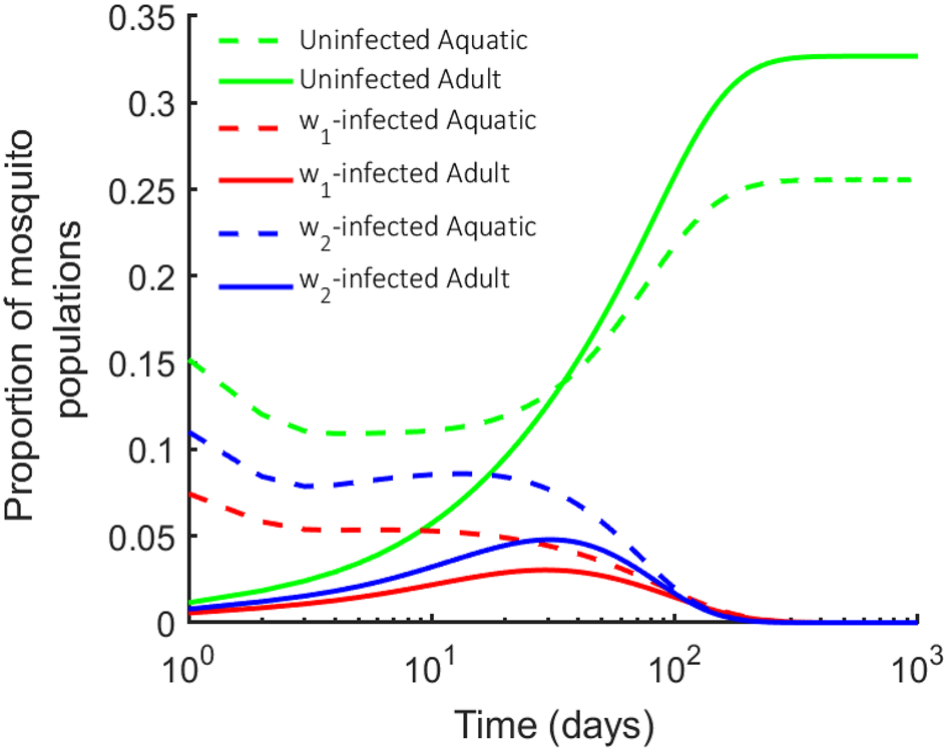
*Wolbachia*-free mosquito equilibrium point $e_u$: The stability conditions for the numerical simulations using $\rho _u = 10$, $\rho _{w_1} = 13$, and $\rho _{w_2} = 11$, leading to $R_{0u}=98>1$, $R_{0w_1|u}=0.66<1$ and $R_{0w_2|u}=0.51<1$. Other parameters used are consistent with Table 1.

Therefore, $e_u$ is locally asymptotically stable if and only if $R_{0u}>1$, $R_{0w_1|u}<1$ and $R_{0w_2|u}<1$ (see Fig. 2).

#### *i-Wolbachia*-infected mosquito population only

Here, we consider the stability conditions for the persistence of a single strain of *i*-*Wolbachia*-infected mosquitoes, and the extinction of all other subpopulations (*j*-*Wolbachia*-infected mosquitoes where $j\ne i$). For the equilibrium point $e_i$, $i\epsilon \{w_1,w_2\}$ to exist, there must be no loss of *Wolbachia* infection ($\sigma _i=0$) and the maternal transmission of *Wolbachia* infection to offspring must be perfect ($\eta _{ii}=1$). From the two-strain *Wolbachia* model (4), the equilibrium point $e_i$, $i\epsilon \{w_1,w_2\}$ is obtained as

$$\begin{aligned} e_i = \left( 0,0,\dfrac{1}{\alpha }\left[ 1-\dfrac{1}{R_{0i}}\right] ,\dfrac{\tau _{i}}{2\alpha \mu _{i}}\left[ 1-\dfrac{1}{R_{0i}}\right] ,0,0\right) \end{aligned}$$

which requires $R_{0i}>1$. Once again we can use the Jacobian method to calculate the invasive reproductive number for the wild-type mosquito population against a background of type *i*-infected mosquitoes; this yields:

6 $$\begin{aligned} R_{0u|i}= & \frac{R_{0u}}{ R_{0i}}\left[ (1-\phi _{ui})+\frac{\rho _{i}}{\rho _u}(1-\eta _{iu})\right] , \end{aligned}$$

where $R_{0u|i}$ in equation (6) is the invasive reproductive number due to uninfected mosquitoes and $\phi _{ui}$ represents the effect of unidirectional CI between an *i*-infected male and an uninfected female.

We can also derive the invasive reproduction number of the other *Wolbachia* strain $j \ne i$ in equation (7) as

7 $$\begin{aligned} R_{0j|i}= & \dfrac{R_{0j}\eta _{ji}}{R_{0i}\eta _{jj}}(1-\phi _{ji}), \end{aligned}$$

where $\phi _{ji}$ represents the bidirectional CI effect between a *j*-infected female and an *i*-infected male. $\eta _{ji}$ and $\eta _{jj}$ denote the proportion of mosquito offspring with *j* infection produced from a *j*-infected female mosquito mating with either an *i*-infected or *j*-infected male mosquito respectively.

Each of the *Wolbachia* strains $i\epsilon \{w_1\text {or}w_2\}$ can establish itself when introduced separately (single *Wolbachia*-infected mosquito introduction) as their equilibrium points are stable^13^, for the parameter values listed in Table 1.

To establish the stability of the *i*-*Wolbachia*-infected population equilibrium point $e_{i}$, we evaluate the Jacobian *J* of the system at $e_{i}$ and compute the characteristic equation in equation (8) as follows:

8 $$\begin{aligned} |J^{e_i}-\lambda I| = (\lambda ^2 + b_1\lambda +b_2) (\lambda ^2 + b_3\lambda + b_4) (\lambda ^2 + b_5\lambda + b_6)=0 \end{aligned}$$

where,

$$\begin{aligned} b_1=\, & \mu _{i} + (\mu _{A_{i}}+\tau _{i}) R_{0i}\\ b_2=\, & \mu _{i} (\mu _{A_{i}}+\tau _{i}) (R_{0i}-1)\\ b_3=\, & \mu _u + \mu _{A_u}+\tau _u\\ b_4= \,& \mu _u (\mu _{A_u}+\tau _u) (1-R_{0u|i})\\ b_5=\, & \mu _{j} + \sigma _{j} + \mu _{A_{j}}+\tau _{j}\\ b_6=\, & (\mu _{j} + \sigma _{j}) (\mu _{A_{j}}+\tau _{j}) (1-R_{0j|i}). \end{aligned}$$

Therefore, the conditions for stability of $e_{i}$ are: $R_{0i}>1$, $R_{0u|i}<1$, $R_{0j|i}<1$ ($i\ne j$).

**Figure 3:**
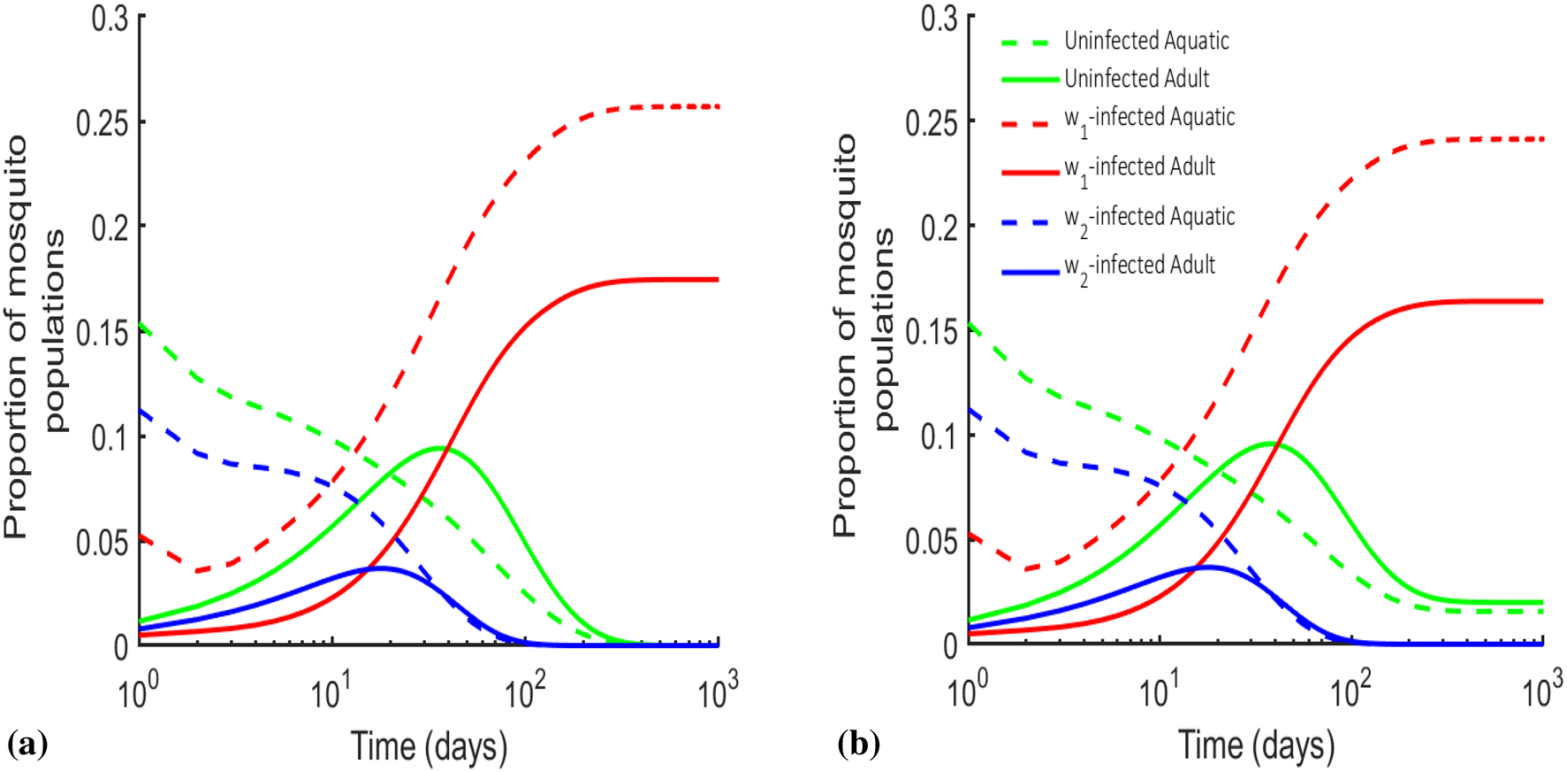
$\mathbf {w_1}$(*w*Au)-infected mosquito equilibrium point $\mathbf {e_{w_1}}$: The graphs show the local stability conditions for $e_{w_1}$. Using $\rho _u = 10$, $\rho _{w_1} = 40$, and $\rho _{w_2} = 11$, **(a)** we set $\eta _{w_1w_1}=1$, $\sigma _{w_1}=0$ and the stability conditions $R_{0w_1}>1$, $R_{0u|w_1}<1$ and $R_{0w_2|w_1}<1$ are satisfied. **(b)** On setting $\eta _{w_1w_1}=0.97$, the $e_{w_1}$ equilibrium point becomes unstable and shifts to $e_{uw_1}$. Other parameters used are consistent with Table 1.

To demonstrate the $e_i$ stability conditions for two specific *Wolbachia* strains, let $w_1=w\text {Au}$ and $w_2=w\text {Mel}$ describe the properties of *w*Au and *w*Mel *Wolbachia* strains respectively. These two *Wolbachia* strains differ in their *Wolbachia* infection retention and CI effect. Therefore, accounting for these differences, the conditions for stability of the *w*Au *Wolbachia*-infected population equilibrium point ($e_{w_1}$) are given as $R_{0w_1}>1$, $R_{0u|w_1}<1$, $R_{0w_2|w_1}<1$ (see Fig. 3a).

Figure 3a showed the stability of the *w*Au-*Wolbachia*-infected population provided that perfect maternal transmission ($\eta _{w_1w_1}=1$) and no *Wolbachia* infection loss ($\sigma _{w_1} = 0$) was observed. But as the maternal transmission becomes imperfect ($\eta _{w_1w_1}<1$), the equilibrium point becomes unstable due to leakage of uninfected mosquitoes as seen in Fig. 3b. Similarly, the same corresponding effect as observed in Fig. 3b is seen if there is an increase in the *Wolbachia* infection loss ($\sigma _{w_1} > 0$).

For the uninfected mosquito population to coexist with *Wolbachia*-infected mosquitoes, one of these two conditions must be satisfied: there must either be a continuous loss of *Wolbachia* infection ($\sigma _{i}>0$), or maternal transmission is imperfect ($\eta _{ii}<1$). Table 2 below provides the CI parameters used in this section.

By these adjustments, we have the coexistence equilibria described below.

**Table 2:** Table showing the effect of CI parameters for different combinations of mosquito crosses (1 = Present, 0 = Absent; UD = unidirectional and BD = bidirectional).

CI Parameters	Mosquito Crosses	CI type	CI effect ($u=$uninfected, $w_1=$*w*Au, $w_1=$*w*Mel/*w*AlbB)
$\phi _{uw_1}$	$F_uM_{w_1}$	UD	0
$\phi _{uw_2}$	$F_uM_{w_2}$	UD	1
$\phi _{w_1w_2}$	$F_{w_1}M_{w_2}$	BD	1
$\phi _{w_2w_1}$	$F_{w_2}M_{w_1}$	BD	0

#### Uninfected and single-infected mosquito populations

Here, we consider the general case of model (4), and in the subsections that follow, special cases are investigated. The general equilibrium point $e_{ui}$ for coexisting uninfected and one of $i\epsilon \{w_1,w_2\}$ infected mosquito populations is

$$\begin{aligned} e_{ui} = \left( \dfrac{2(\beta \mu _{u}-\sigma _{i}) F_{i}^*}{\tau _{u}}, \beta F_{i}^*, \dfrac{2(\mu _{i}+\sigma _i)F_{i}^*}{\tau _{i}}, F_{i}^*,0,0\right) , \end{aligned}$$

where

9 $$\begin{aligned} F_{i}^* = \dfrac{\left( 1-\frac{H}{R_{0i}}\right) \tau _{u}\tau _{i}}{2\alpha ((\mu _{i}+\sigma _i)\tau _{u} + (\beta \mu _{u}-\sigma _i)\tau _{i})}, \end{aligned}$$

and

$$\begin{aligned} H = \dfrac{(1+\beta )}{\left( 1+\frac{\eta _{iu}}{\eta _{ii}}\beta \right) }, \end{aligned}$$

as

10 $$\begin{aligned} a_1\beta ^2 + b_1\beta +c_1 = 0, \end{aligned}$$

where,

11 $$\begin{aligned} a_1= & R_{0i|u}-1 \end{aligned}$$

12 $$\begin{aligned} b_1= & \frac{R_{0i}}{R_{0u}}\left[ \left( \frac{R_{0u}}{R_{0i}}\frac{\sigma _i}{\mu _u}R_{0i|u}+R_{0u|i}\right) -1\right] \end{aligned}$$

13 $$\begin{aligned} c_1= & \frac{\rho _{i}}{\rho _{u}} (1-\eta _{ii}) + \frac{\sigma _i\eta _{ii}}{\eta _{iu}\mu _u}R_{0i|u}. \end{aligned}$$

Therefore, for $e_{ui}$ to exist for any *i-Wolbachia* strain, $\eta _{ii}<1$ or $\sigma _{i}>0$ given the conditions $\beta \mu _{u} > \sigma _i$ and for $H\ge 1$, $\eta _{iu}\le \eta _{ii}\le 1$. To establish stability, $R_{0i}>H\ge 1$, $R_{0i|u} > 1$, $\left( \frac{R_{0u}}{R_{0i}}\frac{\sigma _i}{\mu _u}R_{0i|u}+R_{0u|i}\right) >1$ and $\eta _{ii}<1$ must be satisfied. According to the Routh-Hurwitz criterion for polynomials^50^, $e_{ui}$ with equation 10 is stable if and only if $\{\frac{b_1}{a_1},\frac{c_1}{a_1}\} >0$. Although $e_{ui}$ could exist if $R_{0i|u} < 1$, $\left( \frac{R_{0u}}{R_{0i}}\frac{\sigma _i}{\mu _u}R_{0i|u}+R_{0u|i}\right) <1$, it is unstable as $\frac{c_1}{a_1}<0$. Interestingly, $e_{ui}$ will exist if the *Wolbachia*-infected mosquitoes do not go extinct when introduced into a completely susceptible wild-type mosquito population provided that there is either no perfect maternal transmission of *Wolbachia* infection $\eta _{ii}<1$ or loss of *Wolbachia* infection at high temperature $\sigma _{i}>0$ occured.

The demonstration of the uninfected and specific *Wolbachia*-infected mosquitoes’ existence has been done by^13^, where the authors considered the coexistence of uninfected and *w*Au-*Wolbachia*-infected mosquitoes. The existence conditions in^13^ are consistent with the existent conditions described in this section.

#### $w_1$ and $w_2$ infected mosquito populations

The equilibrium point for coexisting $w_1$ and $w_2$ infected mosquito populations in the absence of wild-type does not exist. This is because there is no dynamical link connecting the population progression of both strains. Although our model described that $w_1$ strain does not induce CI, $w_2$ does. Therefore, these two strains could not coexist in the absence of wild-type mosquitoes as a result of direct offspring competitive exclusion.

We proceed to investigate the three populations existence equilibrium point.

#### Uninfected, $w_1$ and $w_2$ infected mosquito populations

The equilibrium point for the uninfected, $w_1$ and $w_2$ populations will only exist if $R_{0w_1|u}>1$, $R_{0u|w_1}<1$, $R_{0w1|u}>R_{0w2|u}$, $R_{0w_2|w_1}>1$ (see Supplementary file). The last two conditions are incompatible if the maximum proportion of offspring generated via maternal transmission is perfect, i.e., $\textrm{max}\{\eta _{ij}=1\}$. This shows that there is no biologically stable equilibrium, only a temporary coexistence can be demonstrated numerically and this has potential advantages.

**Figure 4:**
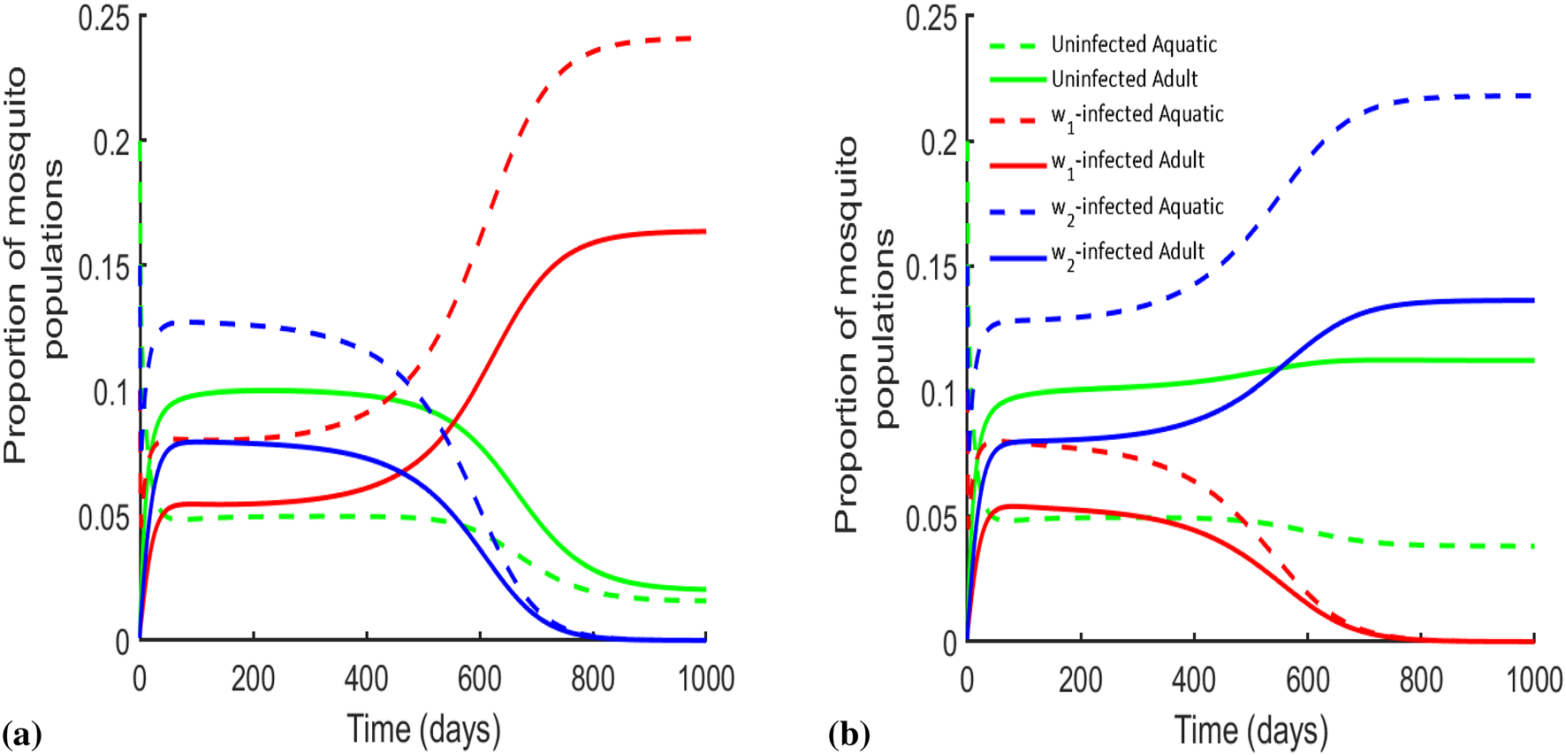
The numerical simulations showing pseudo existence of $\mathbf {e_{uw_1w_2}}$. For $R_{0u},R_{0w_1},R_{0w_2}>1$, using $\rho _u = 10$, $\rho _{w_1}=41.5$ and $\rho _{w_2}=30$, **(a)** Showed that mosquitoes with strains *w*Au (with maternal transmission of $\eta _{w_1w_1}=0.97$), *w*Mel ($\eta _{w_2w_2}=0.97$) and wild-type exist for a time and then one of the *Wolbachia* infected mosquitoes-*w*Mel is eliminated by the other dominating *w*Au-infected mosquito population showing instability. **(b)** Showed that an infinitesimal decrease in the reproductive rate of *w*Au-infected mosquitoes, i.e. ($\rho _{w_1}=41.4$), eliminates *w*Au-infected mosquito population and allows for the coexistence of uninfected and *w*Mel-infected mosquitoes. Other parameters are consistent with Table 1.

For the three (uninfected, *w*Au-infected, *w*Mel-infected) mosquito populations to temporarily exist, we require $R_{0u}, R_{0w_1}, R_{0w_2}>1$ (see Fig. 4). It is observed that, the populations can only exist at most for some time (1-2 years) in this case, however, the dominating *Wolbachia* strain will eventually knock out the other depending on parameters contributing to its invading force or characteristics such as maternal transmission of *Wolbachia* infection, reproductive and loss of *Wolbachia* infection rates (Fig. 4). This is called the founder control as established in^28^. Interestingly, some mathematical and biological implications could be derived between the pseudo-stable times prior to the founder control effect. These implications are elaborated in the next section (Section 5) outlining the tradeoffs between using one and two strains of *Wolbachia* infected mosquitoes to control arboviral infections.

### The trade off between one and two ***Wolbachia*** strains

The competitions between the *Wolbachia* uninfected and infected mosquitoes vary. However, these competitiveness is based on the contributions of CI, per capita reproductive rate and loss of *Wolbachia* to their reproduction rates. First, in the absence of loss of *Wolbachia* infection, two different *Wolbachia* infected mosquitoes that each possess CI (i.e. four out of nine possible mating combinations will induce CI) could be advantageous compared to other paired combinations. In addition, this advantage could also outweigh that of a CI-inducing single *Wolbachia* strain (one out of four possible mating combinations only induce CI). However, this is not the case as the temperature effect will take its course on the *Wolbachia*-infected mosquitoes due to seasonally-varying weather conditions.

**Figure 5:**
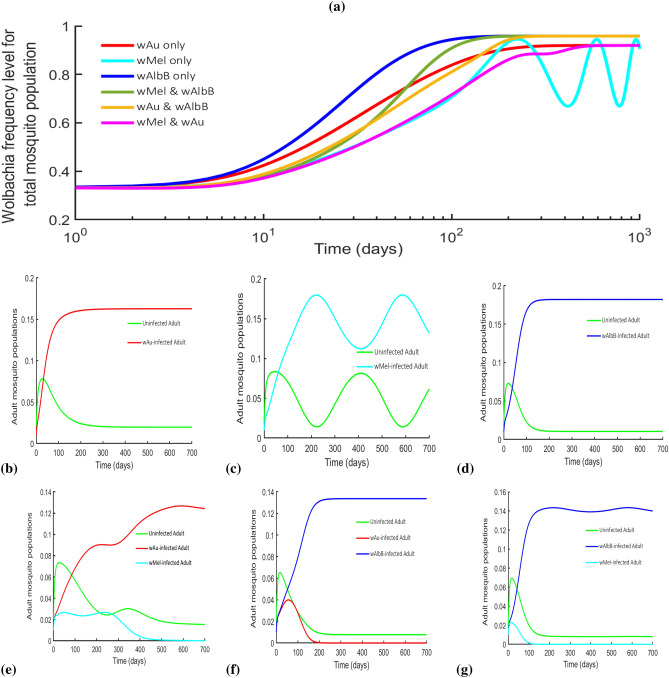
Wolbachia frequency levels for both single and double-strain *Wolbachia*-infected mosquitoes in the presence/absence of CI and *Wolbachia* infection loss. (**a**) Showed the *Wolbachia* frequency levels of both one-strain—*w*Au; *w*Mel; *w*AlbB, and two-strain—*w*Au & *w*AlbB; *w*Au & *w*Mel; and *w*Mel & *w*AlbB *Wolbachia*-infected mosquitoes, accounting for the effects of uni- and bidirectional CIs and the LWI (parameters used can be found in Table 3). **(b)** Showed the adult mosquitoes for *w*Au-*Wolbachia* only competition with the wild type mosquitoes as in *w*Au only *Wolbachia* frequency in **(a)**. **(c)** Showed the adult mosquitoes for *w*Mel-*Wolbachia* competition only with uninfected mosquitoes as in *w*Mel only in **(a)**. **(d)** Showed the adult mosquitoes for *w*AlbB-*Wolbachia* competition only with uninfected mosquitoes as shown in **(a)**. **(e)** Showed the adult mosquito population of the two strain competition of *w*Au and *w*Mel together with wild-type mosquitoes for the *Wolbachia* frequency of *w*Au and *w*Mel in **(a)**. **(f)** Showed the adult populations for uninfected, *w*Au- and *w*AlbB-infected mosquitoes for the *Wolbachia* frequency in **(a)**. **(g)** Showed the adult populations for *w*AlbB- and *w*Mel-infected mosquitoes for the *Wolbachia* frequency in **(a)**.

Therefore, considering the effect of temperature, we model the $\sigma _i$ (the loss of *Wolbachia* infection rates for the $i\epsilon \{w_1,w_2\}$ *Wolbachia* infection) as a function of seasonal variations (with time) for *Wolbachia* loss in equation (14)

14 $$\begin{aligned} \sigma _i(t) = \frac{\sigma _{m_i}}{2}\left( \cos \left( \frac{2\pi t}{365}-\varOmega \right) +1\right) , \end{aligned}$$

where $\sigma _{m_i}$ describes the maximum value of the seasonal fluctuation in the *Wolbachia* loss for the corresponding strains $i\epsilon \{w_1,w_2\}$. $\varOmega$ represents the phase shift of the transcedental function that positions the model with the seasonal variation. Figure 5a shows the *Wolbachia* frequency levels for a single-strain (*w*Au, *w*Mel and *w*AlbB) and a combination of double-strain (*w*Au with *w*Mel, *w*Au with *w*AlbB and *w*Mel with *w*AlbB) *Wolbachia*-infected mosquitoes in the presence/absence of CI and *Wolbachia* infection loss properties. Figure 5a is disintegrated into Figures (b),(c),(d),(e),(f) and (g). Figure 5b describes the single-strain *w*Au-*Wolbachia*-infected mosquito dominance after 7-8 months in the absence of *Wolbachia* heat loss and CI induction. On the other hand, Fig. 5c visualises the effect of LWI on single-strain *w*Mel-*Wolbachia*-infected mosquitoes seasonally over the years despite lack of CI. Figure 5d shows similar dynamics as in Fig. 5b but had a decreased number of wild type mosquitoes and as a result, increased number of *w*AlbB mosquitoes due to lack of CI. For the double-strain (*w*Au with *w*Mel), *Wolbachia*-infected mosquitoes, Fig. 5e shows that the two strains could be maintained before exhibiting the founder control and as such, a gradual dominating strain (*w*Au in this case) knocks out the other (*w*Mel) after 1.4 years. This occured as *w*Mel-mosquitoes with CI continually lose their *Wolbachia* infection due to heat while the non CI-inducing *w*Au-mosquitoes do not, therefore strengthening the fact that the gains from not losing *Wolbachia* infection outweigh those of CI^13^. Further, Fig. 5f shows that the combination of the two (*w*Au and *w*Mel) *Wolbachia* strains would lead to a longer time for dominance to occur as seen in Fig. 5a.

For the other two-strain combinations i.e *w*Au and *w*AlbB, and *w*Mel and *w*AlbB (Fig. 5f,g), there is an increase in the frequency levels of *Wolbachia* compared to *w*Au- and *w*Mel-only strain (as seen in Fig. 5a). However, it was observed that *w*AlbB-only *Wolbachia* strain was able to dominate faster and performed best in all comparisons in terms of having higher affinity to retain *Wolbachia* infections in mosquitoes at high weather temperatures in the absence of CI (Fig. 5a).

Therefore, starting a *Wolbachia* rollout with two strains simultaneously may not be advantageous as the time to dominate the population could be reached faster using a single strain with high *Wolbachia* retention at high temperature in the absense of CI (see Fig. 5).

**Table 3:** Table showing the parameter values used for the effect of CI and LWI on seasonal variation for one and two *Wolbachia* strains.

*Wolbachia* Strain(s)	$\phi _{uw_1}$	$\phi _{uw_2}$	$\phi _{w_1w_2}$	$\phi _{w_2w_1}$	$\sigma _{m_1}$	$\sigma _{m_2}$
**One Strain**						
*w*Au	0	–	–	–	0	–
*w*Mel	1	–	–	–	0.025	–
*w*AlbB	1	–	–	–	0	–
**Two Strains**						
*w*Au & *w*Mel	0	1	1	0	0	0.025
*w*Au & *w*AlbB	0	1	1	0	0	0
*w*AlbB & *w*Mel	1	1	1	1	0	0.025

## Discussion

In this paper we set out to explore the impact of introducing two *Wolbachia* strains simultaneously. Using information on the ecological dynamics of multiple *Wolbachia* strains with various characteristics^1,6,28,29,39^, we were interested in exploring stable co-existence and synergistic effects. We found neither of these. Specifically, we found that the fitter *Wolbachia*-infected mosquito strain would dominate and eliminate the other strain meaning that co-existence would always be temporary. Furthermore, the temporary co-existence did not increase prevalence of *Wolbachia* strains, and either had no impact or reduced prevalence.

Our motivation for examining co-existence was based on the recognition that some studies have shown that a *Wolbachia* strain: *w*Au, does not exhibit either unidirectional or bidirectional cytoplasmic incompatibility (CI)^3,10^. That is, when a *w*Au-infected *Wolbachia* male mosquito is crossed with another strain *Wolbachia*-infected female, they produce offspring with the other *Wolbachia* strain. For this reason, we believed that combining *w*Au with other strains may not interfere with the dynamics of the other strain and could potentially be synergistic. This is particularly so because *w*Au has the positive feature of high heat tolerance, which plausibly may outweigh the lack of CI^13,30^. Therefore, we developed a two-strain general model (4) and tuned the parameters to reflect properties of *w*Au, *w*Mel and *w*AlbB in turn.

Our two-strain general model described the transmission dynamics of uninfected and *Wolbachia*-infected mosquitoes with two different strains (Supplementary Figure S2). We derived the general mosquito-free reproduction numbers and further established the *Wolbachia* invasive reproduction numbers singly for the two strains using the *Wolbachia*-infection free equilibrium point. These invasive reproduction numbers were used to establish the local stability conditions of the equilibrium points and were in line with results from single strain models reported previously^13^. In the general model, we specifically examined *w*Au: with absent CI and good *Wolbachia* retention in heat and we combined this (in our *in silico* model) with *w*Mel & *w*AlbB: CI present in both and loses/retains *Wolbachia* infection in heat, respectively. Considering the transmission dynamics involving these single strains, we established that there was local stability for each of *w*Au-infected and *w*AlbB-infected mosquitoes and that they would dominate uninfected mosquitoes provided there was no loss of *Wolbachia* infections due to high temperature and a complete maternal transmission is exhibited from male and female mosquito crosses with similar strains. However, a single population of only *w*Mel-infected mosquitoes does not exist indefinitely, as uninfected mosquitoes emerge because of loss of infection in this strain at high temperatures.

For each of the strains *w*AlbB and *w*Au-infected mosquitoes, we assume perfect maternal transmission and no heat loss. Under these circumstances there is no stable equilibrium with uninfected mosquitoes. The system dynamically converges to a single-population equilibrium i.e either uninfected or *w*Au/*w*AlbB-only-infected population. This is because, the perfect maternal transmission blocks any leakage of uninfected offspring making the steady state of zero uninfected mosquitoes and 100% *w*Au-infected mosquitoes stable provided its invasive reproduction number is greater than one. In contrast, the coexistence of the uninfected and the CI-inducing *w*Mel-infected mosquitoes exists as the *w*Mel-infected mosquitoes are continuously losing their infections due to high temperature. Under these circumstances, the coexistence with uninfected mosquitoes will continue to exist provided there is *Wolbachia* infection loss. For all three strains, there is a potential uninfected-mosquito only equilibrium if the *Wolbachia*-infected mosquitoes are unable to invade an existing uninfected population (when the invasive reproduction number for *Wolbachia* infected mosquitoes is less than one).

While co-existence of a single strain of *w*Mel and uninfected mosquitoes is stable (via loss of *Wolbachia* infection in mosquitoes), we found no such stability point for two different strains of *Wolbachia*-infected mosquitoes. Nevertheless, we showed through numerical simulation that under plausible parameter ranges, *Wolbachia* strains may coexist for a year or two. However, this co-existence is always temporary and cannot attain stability as one strain will dominate the other to exclusion. Once a population of mosquitoes is present in the population, it becomes harder for species to invade, and the founder strain will exclude any competing strain^28^. We showed numerically that before hitting founder control, the two different *Wolbachia*-infected mosquito populations coexisted for some time, providing some hope of establishing a synergistic effect. However, our study showed that introducing two strains of *Wolbachia* simultaneously could neither fast track the time to *Wolbachia* dominance in the wild-population nor increase the *Wolbachia* prevalence compared to a single *Wolbachia* strain release of the fitter strain (in our context *w*AlbB). This was also true for the combination of *w*Au and *w*Mel, with *w*Au as a single strain out-performing the introduction of both strains simultaneously.

Our work therefore leads to the recommendation of rolling out one-strain of *Wolbachia*-infected mosquitoes with optimal characteristics (high *Wolbachia* infection retention at high temperature, high maternal transmission and complete CI) rather than attempting mixed strain rollouts.

## Supplementary Information

Supplementary Information.

## Data Availability

All data generated or analyzed during this study are included in this published article [and its supplementary information files].
