# Supplementary material for: Modelling the ecological dynamics of mosquito populations with multiple co-circulating Wolbachia strains
Source: Sci Rep. 2022 Dec 2;12:20826. doi: 10.1038/s41598-022-25242-x (PMC9718785; doi:10.1038/s41598-022-25242-x)
Supplement: Supplementary file 1 — Supplementary Information. [file 41598_2022_25242_MOESM1_ESM.pdf]

Supplementary information

Mosquito-free equilibrium

Here, we want to establish the mosquito-free equilibrium point is $e_0 = \{0,0,0,0,0,0\}$. This equilibrium point is not naturally realistic as it is trivial. However, useful inferences could be achieved via the interactive model dynamics by investigate each of the three (u, w_1, w_2) mosquito populations and how they would perform when the others are absent.

From the system of equations (2), we derive the reproductive number of the uninfected mosquito population R_{0u} and those with each infection status R_{0i} as:

$$R_{0u} = \frac{\rho_u(1-\phi_{uu})\psi\tau_u}{\mu_u(\mu_{A_u}+\tau_u)} = \frac{\rho_u\psi\tau_u}{\mu_u(\mu_{A_u}+\tau_u)},$$
$$R_{0i} = \frac{\rho_i\eta_{ii}(1-\phi_{ii})\psi\tau_i}{(\mu_i+\sigma_i)(\mu_{A_i}+\tau_i)} = \frac{\rho_i\eta_{ii}\psi\tau_i}{(\mu_i+\sigma_i)(\mu_{A_i}+\tau_i)},$$

for $i\epsilon\{w_1, w_2\}$ and where $\phi_{uu} = \phi_{ii} = 0$. This is because CI does not affect the matings between two equal pairs of mosquitoes. Additionally, we assume that $\psi = 1/2$ as the ratio of female to male mosquitoe is approximately 1:1. Irrespective of the *Wolbachia* strains involved in the *Wolbachia* single or double rollout dynamics, the basic mosquito reproductive number is independent of CI, however, may be dependent on the *Wolbachia* infection loss σ_i at high temperatures.

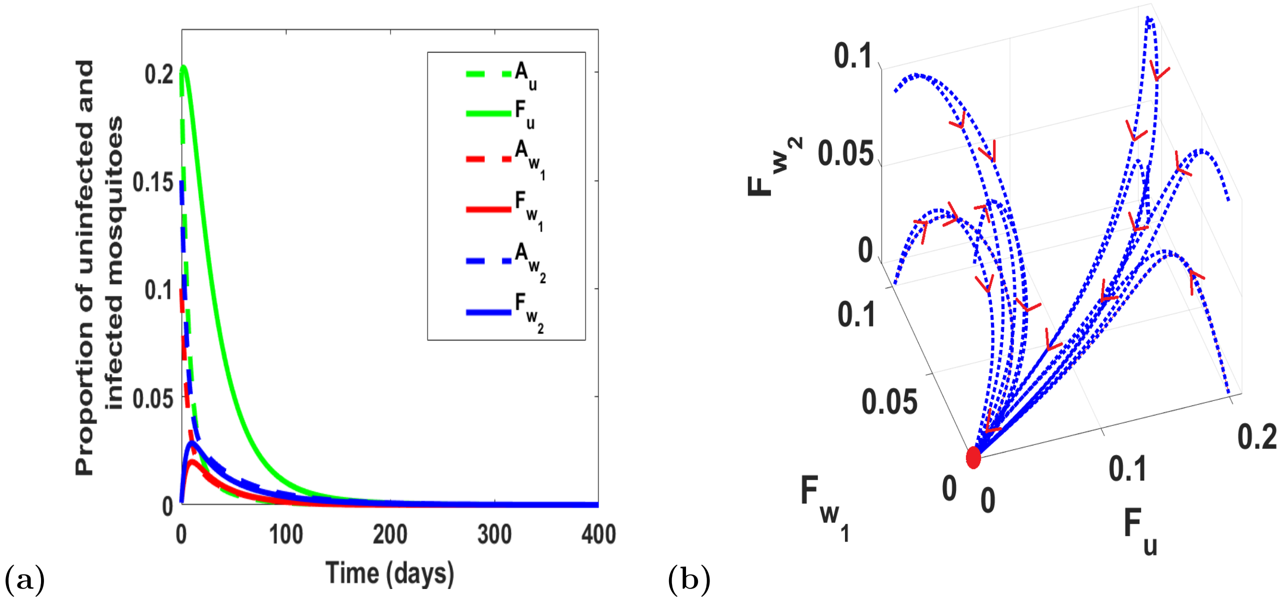

Figure S1: **No mosquito equilibrium point.** (a) Shows the stability of e_0. We set $\rho_u = 0.01$, $\rho_{w_1} = 0.12$, $\rho_{w_2} = 0.15$ and $R_{0u} = 0.098$, $R_{0w_1} = 0.6080$, $R_{0w_2} = 0.6995$. (b) Numerical simulations showing the trajectories (in blue dashed curves with red arrows) in the (F_u, F_{w_1}, F_{w_2}) coordinates for when $\max[R_{0u}, R_{0w1}, R_{0w2} < 1]$. The red ball point represents the stability point, i.e. $(F_u, F_{w_1}, F_{w_2}) = (0,0,0)$

If these reproductive numbers $R_{0u}, R_{0wi} < 1$, the populations of both uninfected and $i\epsilon\{w_1, w_2\}$ infected mosquitoes will go extinct. Otherwise, the mosquito populations will persist (see figure S1).

Three mosquito populations (uninfected, w_1 and w_2 infected mosquitoes)

The equilibrium point for the uninfected, w_1 and w_2 populations is

$$e_{u,w_1,w_2} = \left(\frac{2(\mu_u - \sigma_{w_1} f(F_u^*) - \sigma_{w_2} g(F_u^*))}{\tau_u}, F_u^*, \frac{2(\mu_{w_1} + \sigma_{w_1}) f(F_u^*)}{\tau_{w_1}}, f(F_u^*), \frac{2(\mu_{w_2} + \sigma_{w_2}) g(F_u^*)}{\tau_{w_2}}, g(F_u^*) \right).$$

In the presence of any two competing strains $i\epsilon\{w_1 \ (w\text{Au}),\ w_2 \ (w\text{Mel}/w\text{AlbB})\}$, the effect of CI is absent between crosses $F_i M_u$ and $F_i M_i$ (i.e. $\phi_{iu} = \phi_{ii} = 0$). In the case of the uninfected and $i\epsilon\{w_1, w_2\}$ different *Wolbachia*-infected mosquito populations, we would like to consider two contrasting *Wolbachia* strains, say wAu and wMel together with the uninfected mosquito populations. Apart from wAu, which has no CI effect, most (if not all) *Wolbachia* strains, have similar characteristics but may differ in *Wolbachia* infection retention at high temperature. As such, we consider the parameters guiding the wAu as $\sigma_{w_1} = 0$ and for wMel we use σ_{w_2} to adjust for the differences in the other strains relating to wMel such as wAlbB/wMelPop/wPip. The effect of unidirectional and bidirectional CI are also defined by $\phi_{uw_1} = \phi_{w_2w_1} = 0$ and $\phi_{uw_2} = \phi_{w_1w_2} = 1$.

On solving the $i\epsilon\{w_1, w_2\}$ infected compartments in (2), we have

$$F_u^* = \frac{F_{w_1}^* R_{0w_1}(R_{w_2|w_1} - 1) + F_{w_2}^* R_{0w_2}}{R_{0u}(R_{0w_1|u} - R_{0w_2|u})} = m(F_{w_1}^*, F_{w_2}^*). \tag{1}$$

For $F_{w_1}^*$ and $F_{w_2}^*$, we have that, on solving the uninfected and w_1 infected compartments in equations (2),

$$F_{w_2}^* = \frac{F_u^* \left(F_u^* \mu_u R_{0u}(R_{w_1|u} - 1) - F_{w_1}^* \mu_u R_{0w_1}(R_{0u|w_1} - 1) \right)}{F_u^* \left(\sigma_{w_2} R_{0w_1|u} + \frac{\rho_{w_2}}{\rho_u}(1 - \eta_{w_2u})\mu_u \right) R_{0u} + F_{w_1}^* \left(\sigma_{w_2} R_{0w_1} + \frac{\rho_{w_2}}{\rho_u}(1 - \eta_{w_2w_1})\mu_u R_{0u} \right)} = n(F_u^*, F_{w_1}^*). \tag{2}$$

Rearrange (1) to make $F_{w_2}^*$ the subject and equate to (2), we obtain:

$$F_{w_1}^* = \frac{F_u^* R_{0u} \left((R_{w_1|u} - R_{0w_2|u}) - F_u^* R_{0w_2} \mu_u (R_{0w_1|u} - 1) \right)}{F_u^* R_{0w_1} \left(R_{0w_2}(1 - R_{0u|w_1})\mu_u + R_{0u}(R_{0w_2|w_1} - 1) \left(\frac{\rho_{w_2}}{\rho_u}(1 - \eta_{w_2u})\mu_u + \sigma_{w_2} R_{0w_1|u} \right) \right) - \sigma_{w_2} R_{0w_1} - \frac{\rho_{w_2}}{\rho_u}(1 - \eta_{w_2w_1})\mu_u R_{0u}} = f(F_u^*) \tag{3}$$

Now, solving the uninfected and w_2 infected compartments in equations (2), we obtain

$$aF_{w_1}^{*2} + bF_{w_1}^* + c = 0 \tag{4}$$

where

$$\begin{aligned} a &= \left(\mu_u F_u^* R_{0u|w_1} R_{0w_1} + F_{w_2}^* (1 - \eta_{w_2w_1}) \frac{\rho_{w_2}}{\rho_u} R_{0u} \right) \\ b &= F_u^* R_{0u} \left(\mu_u F_u^* + (1 - \eta_{w_2u}) \frac{\rho_{w_2}}{\rho_u} F_{w_2}^* \right) + \eta_{w_2w_1} \frac{\rho_{w_2}}{\rho_{w_1}} F_{w_2}^* R_{0w_1} \left(\sigma_{w_2} F_{w_2}^* - \mu_u F_u^* \right) \\ c &= \left(F_{w_2}^* + \eta_{w_2u} F_u^* \right) \left(\sigma_{w_2} F_{w_2}^* - \mu_u F_u^* \right) F_{w_2}^* \end{aligned}$$

Rearrange (1) to make $F_{w_1}^*$ the subject and substitute into (4), we obtain a real solution

$$F_{w_2}^* = F_u^* \frac{R_{0u|w_1} R_{0w_1} R_{0w_2} \mu_u \rho_u}{(R_{0w_2|w_1} R_{0w_1} - R_{0w_2}) R_{0u} \rho_{w_2}} = g(F_u^*) \tag{5}$$

Substitute (3) and (5) into (1), we obtain:

$$F_u^* = h(f(F_u^*), g(F_u^*)). \tag{6}$$

Therefore, making F_u^* the subject, we obtain:

$$F_u^* = \frac{P}{Q}. \tag{7}$$

Where,

$$\begin{aligned}
P &= \rho_{w_2} R_{0u}(1-\eta_{w_2 w_1})(p_1+p_2)+p_3 \\
Q &= R_{0w_1}\left(\rho_{w_2} R_{0u}^2(1-\eta_{w_2 w_1})(q_1+q_2+q_3)+\mu_u\rho_u R_{0w_1} R_{0w_2} R_{0u|w_1}(q_4+q_5)\right) \\
p_1 &= \mu_u\rho_{w_2}(1-\eta_{w_2 w_1}) R_{0u}^2 (R_{0w_1|u}-R_{0w_2|u}) \\
p_2 &= \rho_u R_{0w_1}(\mu_u^2 R_{0u|w_1} R_{0w_2}+R_{0u}(R_{0w_1|u}-R_{0w_2|u})(\sigma_{w_2}+(R_{0w_2|w_1}-1))) \\
p_3 &= \mu_u\rho_u^2\sigma_{w_2} R_{0w_1}^2 R_{0w_2} R_{0u|w_1} \\
q_1 &= \mu_u\rho_u R_{0w_2}(1-R_{0u|w_1}(R_{0w_1|u}-R_{0w_2|u})) \\
q_2 &= \mu_u\rho_u R_{0w_2}(R_{0w_2|w_1}(R_{0w_1|u}-1)-R_{0w_2}) \\
q_3 &= R_{0u}(R_{0w_1|u}-R_{0w_2|u})(R_{0w_2|w_1}-1)(\mu_u\rho_{w_2}(1-\eta_{w_2 u})+\rho_u\sigma_{w_2} R_{0w_1|u}) \\
q_4 &= \mu_u\rho_u R_{0w_2}(1-R_{0u|w_1}) \\
q_5 &= R_{0u}(R_{0w_2|w_1}-1)(\mu_u\rho_{w_2}(1-\eta_{w_2 u})+\rho_u\sigma_{w_2} R_{0w_1|u}).
\end{aligned}$$

For $e_{uw_1w_2}$ to exist, $R_{0w_1|u} > 1$, $R_{0u|w_1} < 1$, $R_{0w1|u} > R_{0w2|u}$, $R_{0w_2|w_1} > 1$.

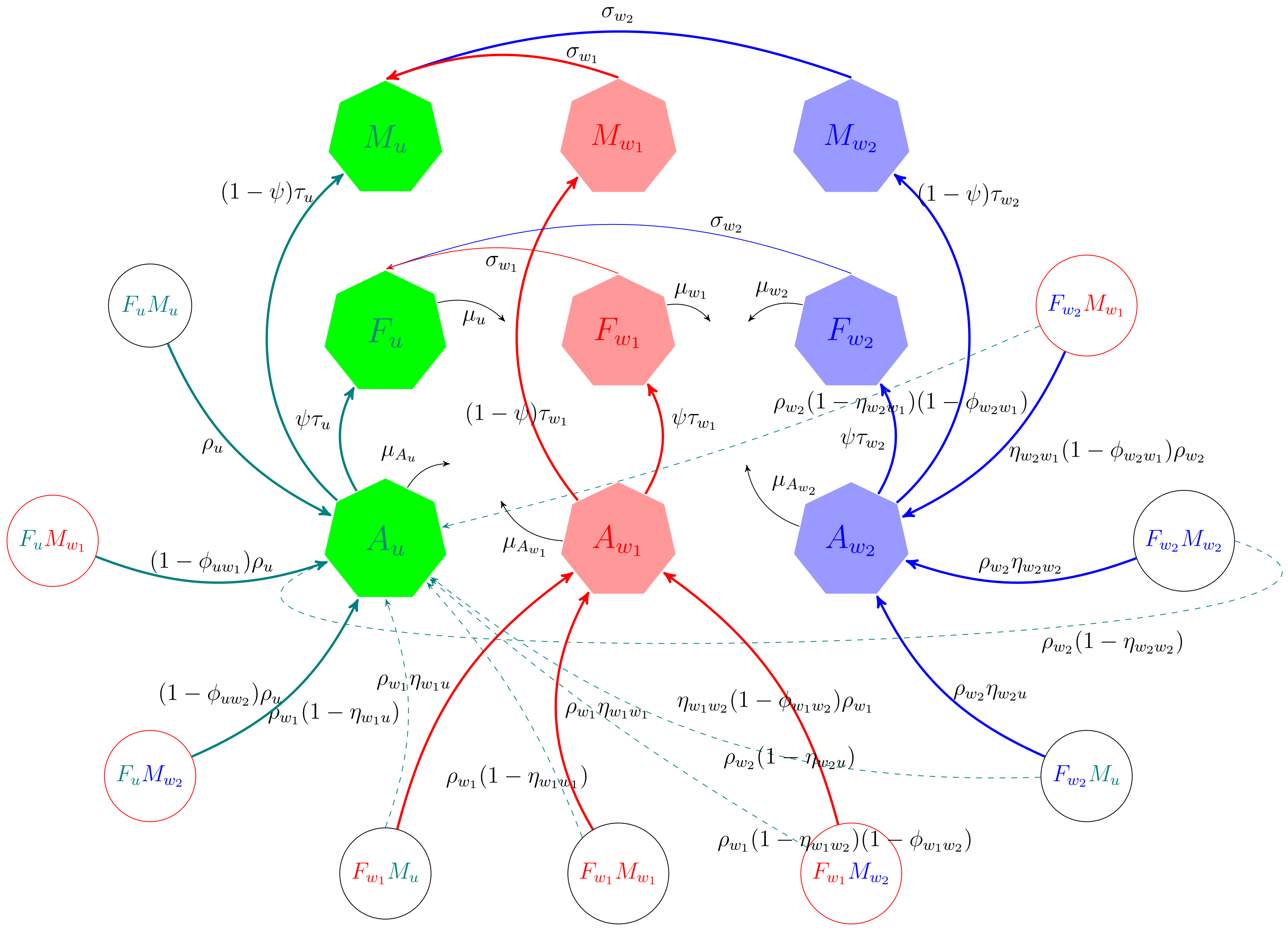

Figure S2: **General model formation schematic of Mosquito-*Wolbachia* dynamics between uninfected mosquitoes and *Wolbachia*-infected mosquitoes with strains $\mathbf{w_1}$ and $\mathbf{w_2}$.** The green red and blue represent the uninfected, w1-*Wolbachia*-infected and w2-*Wolbachia* infected mosquito populations respectively. The solid lines describe how the populations progressed and the dashed lines represent the imperfect maternal transmission (IMT). The $\phi_{i,j}, (i = u, w_1, w_2, j = w_1, w_2)$, represent the induction of cytoplasmic incompatibility (CI), inhibiting the production of offspring. See Table 1 for the symbols' descriptions

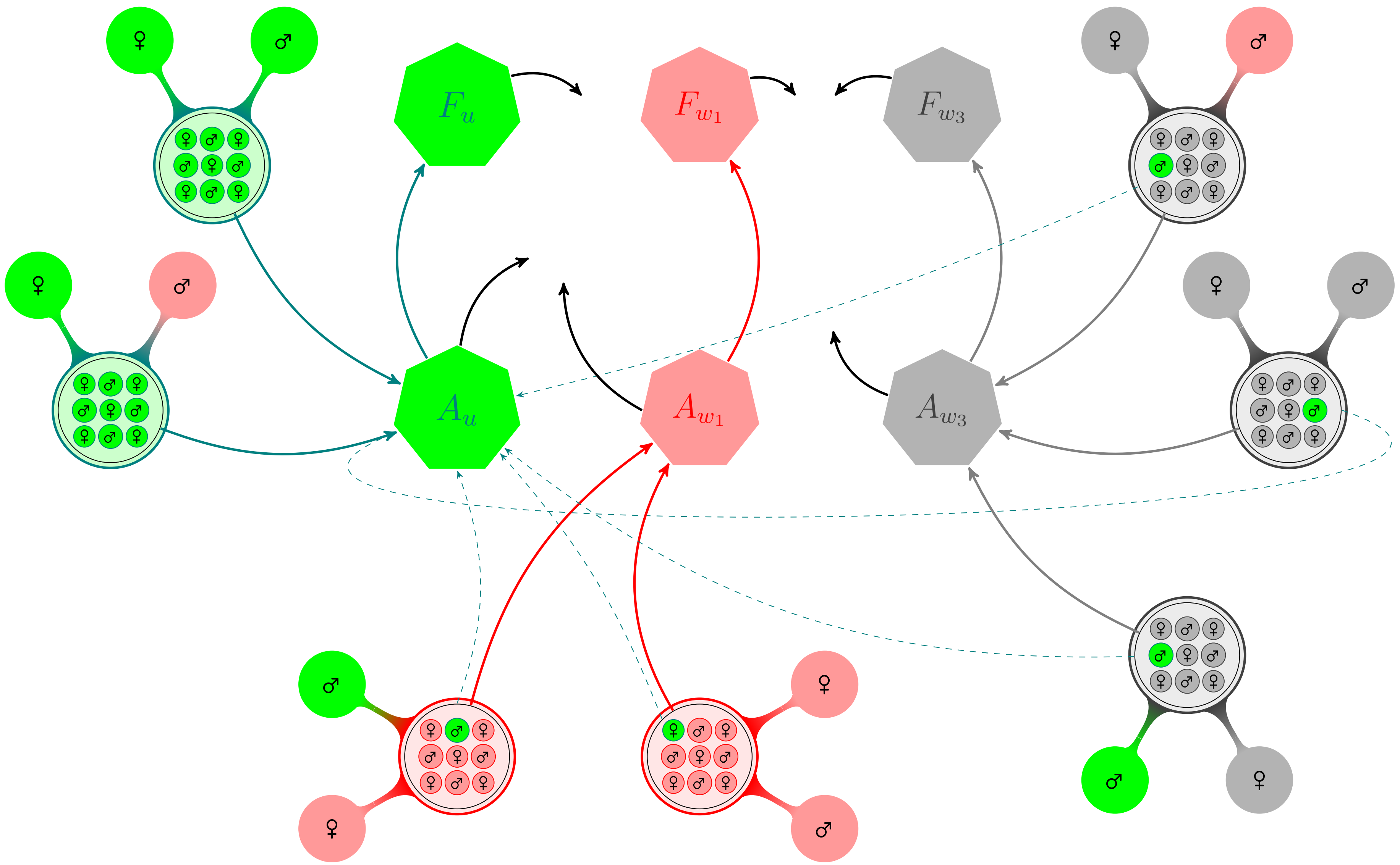

Figure S3: **Reduced ($M = F$) and adjusted model formation schematic of Mosquito-*Wolbachia* dynamics between uninfected mosquitoes and *Wolbachia*-infected mosquitoes with strains $\mathbf{w_1}$ (*w*Au-like) and $\mathbf{w_3}$ (*w*AlbB-like).** The green, red, and grey represent the uninfected, *w*Au-*Wolbachia*-infected and *w*AlbB-*Wolbachia* infected mosquito populations respectively. The solid lines represent the population progression and the dashed lines indicate the imperfect maternal transmission (IMT). The black colour represents deaths. The cytoplasmic incompatibility (CI) induction which inhibits the production of offspring has been adjusted where required.

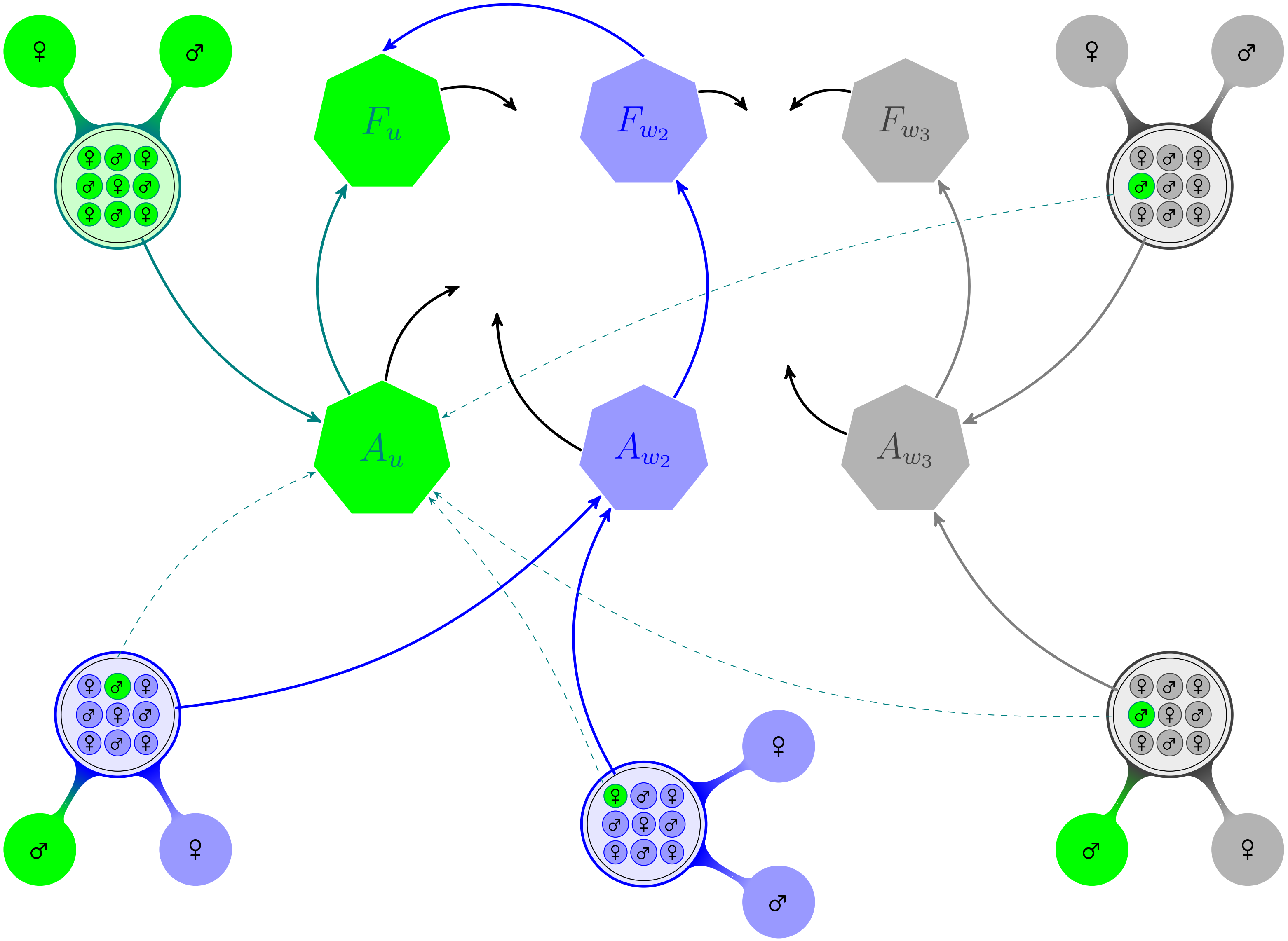

Figure S4: **Reduced ($M = F$) and adjusted model formation schematic of Mosquito-*Wolbachia* dynamics between uninfected mosquitoes and *Wolbachia*-infected mosquitoes with strains $\mathbf{w_2}$ (*w*Mel-like) and $\mathbf{w_3}$ (*w*AlbB-like)**. The green, blue, and gray represent the uninfected, *w*Mel-*Wolbachia*-infected and *w*AlbB-*Wolbachia* infected mosquito populations respectively. The solid lines represent the population progression and the dashed lines indicate the imperfect maternal transmission (IMT). The black colour represents deaths. The cytoplasmic incompatibility (CI) induction which inhibits the production of offspring has been adjusted where required.
